# Improving health and social care services for slum-dwelling older adults: Perspectives of health professionals

**DOI:** 10.3389/fpubh.2022.988076

**Published:** 2022-10-10

**Authors:** Priscilla Yeye Adumoah Attafuah, Irma H. J. Everink, Christa Lohrmann, Aaron Asibi Abuosi, Jos M. G. A. Schols

**Affiliations:** ^1^School of Nursing and Midwifery, University of Ghana, Accra, Ghana; ^2^Department of Health Services Research and Care and Public Health Research Institute, Maastricht University, Maastricht, Netherlands; ^3^Department of Nursing, Institute of Nursing Science, Medical University of Graz, Graz, Austria; ^4^Health Services Management Department, University of Ghana Business School, Accra, Ghana; ^5^Department of Family Medicine and Care and Public Health Research Institute, Maastricht University, Maastricht, Netherlands

**Keywords:** health care service, social care services, slum dwellers, older adults, health professionals

## Abstract

**Background:**

Besides confronting the challenges of a growing older population, developing countries are dealing with limited resources and infrastructure, to ensure good health and social care services. One of these developing countries facing these challenges is Ghana. The healthcare system in Ghana currently does not have specialized geriatric services and is funded through the National Health Insurance Scheme (NHIS), private insurance companies and an out-of-pocket expenditure system. Social care services are important in improving Quality of Life (QoL) as it helps in building and strengthening relationships while also keeping slum-dwelling older adults active. There are various challenges with the health and social care of older adults in slums and practical ways to improve these have not been explored among the providers of this care.

**Aims:**

This study, therefore, aimed to explore (1) the views of health professionals on older slum-dwelling adults' health and social care needs, access, and use, and (2) recommendations for improving access to health and social care services among slum-dwelling older adults.

**Method:**

A qualitative exploratory descriptive approach was used among health professionals by conducting a focus group discussion (FGD) and interviews. A semi-structured interview guide was used to collect data from each participant.

**Results:**

A total of 27 participants took part in the study. In the analysis of transcripts, 3 themes and 14 subthemes were conceptualized. Financial difficulties, queueing issues, distance to health facilities, health illiteracy and negative attitude of health professionals were identified as some barriers to the utilization of formal healthcare services. Social care services were described as non-existent, not structured, and having limited resources to cater for attendants. The health professionals also provided recommendations for improvement.

**Conclusion:**

Health professionals in this study discussed barriers to access and use of health and social care services. Addressing these barriers is essential to improve the use of formal health and social care services and diminish health inequity among older adults.

## What is known

- Barriers such as financial challenges, health illiteracy, long distances to services, long waiting times in healthcare facilities, and poor attitude of health professionals.

## What study adds

- Formal social care services appeared to be next to non-existent.
- Available social care services are not structured.
- Recommendations for government agencies and NGOs to partner to provide formal social care services.
- Considerations for telehealth in slums to provide services for older adults.

## Introduction

Adequate and sufficient healthcare and social services contribute to the quality of life (QoL) of older individuals and need to be present to promote healthy living (1, 2). Globally, societies are aging, and this is also the case for developing countries. Besides facing the challenges of a growing older population, developing countries are dealing with limited resources and infrastructure, to ensure good health and social care services. One of these developing countries facing these challenges is Ghana.

Ghana is a lower-middle-income country (according to the World Bank country classifications) with a total population of about 29.6 million inhabitants. Approximately 976,000 (3.2%) of the population are older adults (60 years and over). It was projected in 2013, that Ghana's older adult population will reach 2.5 million (7.2%) by 2025 and 6.3 million (11.2%) by 2050. As aging is often associated with frailty, multimorbidity and handicaps (3, 4), many older adults need help and support.

The healthcare system in Ghana currently does not have specialized geriatric services and is funded through the National Health Insurance Scheme (NHIS), private insurance companies and an out-of-pocket expenditure system (5–7). The NHIS is open to all Ghanaians, and one must be registered to enjoy its benefits. For formal workers, registration in the NHIS is free. Non-formal and private workers must pay a fee for NHIS registration and must renew their membership every year (5–7). As has been outlined by various authors, this is one of the deficits of the NHIS (8–10). Many people living in slums are part of this group of non-formal workers. Due to their lack of financial resources, they are often unable to pay the NHIS fee, and therefore, they often lack health care insurance. What also makes healthcare insurance less attractive is that most of the health conditions experienced by older adults are not being covered. For example, most cancer treatments, hearing and optic aids, as well as prostheses and physiotherapy, are not paid for by the NHIS (11, 12). As a consequence, many older adults living in Ghanaian slums do not have healthcare insurance. As they are often unable to pay for healthcare themselves, they usually do not receive the required care and support they need and are highly dependent on relatives.

A population in Ghana particularly vulnerable to this lack of access to care and support are older adults living in slums. Slums are characterized by uneven walkways, poor ventilation, improper housing structures, lack of water supply and poor sanitary conditions. As postulated by previous studies, slum environments as mentioned above may negatively impact the health and QoL of older adults (13–16). Therefore, older slum-dwellers in Ghana represent a particularly vulnerable population, who need adequate formal healthcare services.

However, access and utilization of formal healthcare services for Ghanaian older slum dwellers are problematic. Next to the already mentioned problems related to the insurance scheme, the first reason for this are the financial constraints of older adults in slums. Older adults living in slums used to be either farmers, fishermen or petty traders in their working lives and these professionals do not benefit from any formal pension schemes (9). Therefore, at 60 years and over, they possibly experience financial difficulties if there is no continual financial support from their jobs or relatives.

Largely, the services are not free for older adults below the age of 70 years. Considering that the general life expectancy is 64 years old, there is only a small group (those who live above 70 years), benefiting from these free healthcare services (11). As stated earlier, the NHIS does not reimburse health facilities for services provided for most health conditions of older adults as it is not part of the insurance package. Therefore, a large group (older adults between 60 and 69 years old) still must pay for services that are not affordable for them (9).

The second reason for lack of access and utilization is that slums are usually situated rather far away from formal health care services (17, 18) and this is also the case in Ghana. As described in a study by Attafuah et al. (under review) on the health and social needs of older adults living in slums, participants stated that health facilities are not in the vicinity and make it difficult for patronage. Proximity to formal health care has been reported to be one of the hindrances to healthcare utilization (17, 18), not only in Ghana (19, 20) but also in other countries like Iran (21), Dhaka (22), and Indonesia (23).

The third reason is that older slum dwellers are often health-illiterate (24, 25). By health literacy, we mean the extent to which these older adults have the skills and available resources to access, recognize, consider, and use health information and services to make informed decisions regarding their health (26). As Nutbeam (27) explained, health literacy influences an individual's health behavior and is crucial to empowerment and a good QoL (28–30). The health literacy of older adults in slums is mostly very poor, which makes older adults' resort to spiritual healing. As described by Attafuah et al. (under review) most older adults living in Ghanaian slums trust traditional medicine more than evidence-based practices provided by formal care services.

The last reason for the lack of healthcare use is older adults argue that it seems as if health professionals in Ghana, often have a bad attitude toward older adults (30, Attafuah et al., under review). Not giving priority to older adults, not addressing them politely, and talking harshly to them were some bad attitudes and behavior mentioned by slum-dwelling older adults. All these factors taken together influence the access and use of health services by older slum dwellers negatively (20, 21, 31, 32). Therefore, it is important to see how the healthcare needs of the older slum dwellers can be met more appropriately, and how access to care can be improved.

Besides looking at the use of and access to healthcare services, it is also important for slum-dwelling older adults to be able to use social services. Social services such as counseling, health education, interaction with peers, and engaging in mind development games are important and meaningful for older adults and could be provided at a center in the community. Social care services are important in improving QoL as it helps in building and strengthening relationships while also keeping slum-dwelling older adults active. However, social services are few to non-existent in slums in Ghana (33, 34). A few day-care centers have been opened in some parts of Accra, the capital city. However, the fees that must be paid to make use of these services are not affordable to older adults in the slums.

A previous study by Attafuah et al. (under review), showed older slum-dwelling adults' perceptions of their health and social needs. Following up on that study, this study is seeking the views of professional caregivers regarding the current and future access to health and social care services of these older adults.

This study, therefore, aimed to explore (1) the views of health professionals on older slum-dwelling adults' health and social care access and use, and (2) recommendations for improving access to health and social care services among slum-dwelling older adults.

## Methods

### Study design

This study used a qualitative exploratory descriptive approach by conducting a focus group discussion (FGD) and interviews.

### Study setting

Two private care homes and two hospitals located close to slums in Accra were purposively selected for this study. The care homes were chosen because of the social services they provide to possibly meet the social care needs of slum-dwelling older adults. The hospitals are the main providers of formal healthcare in the region.

Care homes in Ghana provide daycare services for older adults, for instance when relatives are going to work. Professionals working in these care homes are managers, registered general nurses, social care workers and health care assistants. They mostly provide services at home, i.e., community-based where they keep the older adult company and assist in laundry, and cooking among others. They rarely offer in-house residency services. With in-house daycare services, older adults gather for social activities, such as Christmas parties, indoor games on holidays and health talks.

The hospitals are both district hospitals with a 50–60 bed capacity. There are no specialized geriatric units in either hospital, but they provide medical and surgical services to older adult patients. Professionals working here include general nurses, community health nurses, public health nurses, midwives, and medical doctors. General nurses are usually in the outpatient department and the admission wards while the community and public health nurses carry out home visits to older adults in slums within the communities. Sometimes these home visits are conducted based on referrals from the general nurses.

### Participants

To be included in this study, the following inclusion criteria were used: (1) being a registered general nurse, a community health nurse, a social worker, or a nurse manager, (2) being employed in one of the four health care facilities selected for this study, (3) having at least one year of working experience in their respective health professions, and (4) having cared for at least one slum-dwelling older adult. Additionally, participants should be able to communicate in English or Twi (a local Ghanaian language). Registered midwives were excluded.

### Recruitment

To recruit participants, the first author PA contacted hospital managers by telephone to ask if they had staff members who were eligible according to the inclusion criteria. If this was the case, PA received telephone numbers to contact these staff members. The contact details of care home providers were taken from the internet. The managers recruited other health professionals from their facilities for the study. The invited professionals were contacted in person, *via* telephone, and met at a date and time convenient for the professionals for the interview. After informed consent was given, an appointment was made for an interview. Interviews were held either at the facility or in the community.

### Study instrument

A semi-structured discussion/interview guide was used to collect data from each participant. The interview questions were classified into two sections: the first section focused on personal and professional information such as the participant's age, sex, work position, and work experience, and the second section was designed by the authors, based on literature to explore health professionals' views on slum-dwelling older adults' access to health and social care, facilitators, and barriers of care and recommendations on ways of improving healthcare and social services of these older adults. Questions in the discussion/interview guide included: (1) How do you perceive the current health and social services of slum-dwelling older people? (2) How would you describe older adults living in slums accessibility and use of hospital services/community care services/ access to aged care services? (3) What social care services are available in and around Teshie and Madina? (4) How can we improve accessibility and usage of health and social care services among slum-dwelling older adults? During the discussion/interview, additional open-ended questions were asked to enable deeper exploration of the issues. Participants were made to further describe their responses and obtain additional data using probing questions (35, 36). The guide was developed following the objectives of the study and the results of a previous study by the authors (Attafuah et al., under review). Expert consultations were done with IE, JS, and CL to ensure that the interview guide reflected the objectives of this study. The interview guide was pretested on two health professionals (a community health nurse and a general nurse) in a district hospital in the Greater Accra region to identify ambiguous questions and clarify them. This ensured the content validity of the interview guide. There was no need to change any questions after the pre-test. Participants involved in pretesting were not included in the final analysis of the data.

### Data collection procedure

Data were collected between March and April 2022 using face-to-face audio-recorded interviews and one focus group discussion (FGD), all led by PA and supported by a research assistant, who tape-recorded the discussions/interview for between 50 min and 1 h. Only one FGD was done because, during the initial FGD, it was observed by the researchers that some participants felt intimidated by their senior colleagues for fear of victimization despite reassurances from the interviewer. Also, it was very difficult to get most categories of health professionals at the same time for a discussion. Individual interviews were the preferred option in most facilities.

At the start of the interview, the purpose of the study was explained orally and participants who agreed to participate were given an informed consent form to sign. The interviews were carried out in private offices at the health center or aged care center. For interviews with community health nurses, interviews were carried out in the hospital or their Community Health Planning and Services (CHPS) zones. Two interviews were conducted in Twi mixed with English as the participants jokingly said they don't understand some of the questions if stated in English. The rigor of the data collection process was ensured throughout the conduct of interviews and FGD. The credibility of participants was established through the method and analyst triangulation.

### Ethical considerations

Ethical clearance (No. 37MH-IRB IPN 29/2022) was obtained from the Institutional Review Board of the 37 Military Hospital. Also, permission from the municipal assemblies of the study sites was obtained. Informed consent from individuals was sought. Additionally, information on voluntary participation and the right to withdraw from the study at any point were shared with all participants. Confidentiality and privacy were guaranteed as pseudonyms were used. Anonymity was maintained by assigning participants with special codes and confidentiality was also maintained by making sure all audio tapes, transcribed data, field notes, and documented information given by the participants were stored and data encrypted. Access to the data is restricted to the research team alone.

### Data management, analysis, and reporting

Interviews and discussions were transcribed verbatim by a research assistant and proofread by the first author to ensure the accuracy of the accounts of the health professionals thereby enhancing the reliability of the findings. Data analysis took place after the first author checked the audio recording and transcriptions. The data were processed using Atlas ti software version 9. A reflexive thematic analysis procedure from the critical realist and constructionist point of view was followed for the analyses of the data. Critical realists believe there is a reason why slum-dwelling older adults (from Attafuah et al., under review) expressed varying perceptions about health professionals based on their observations. Only health professionals can explain the “real” reason. Hence an interaction with health professionals themselves will better explain their actions as observed by the slum-dwelling older adults and provide recommendations to curtail them. Taking the critical constructionist view was to critically identify various ways of improving health and social care services through a discourse with the health professionals.

The first author familiarized herself with the data by listening to the audio and reading the transcribed data. Following the transcription, two experts who were fluent in both the local language (Twi) and English languages translated those transcribed Twi interviews to English adhering to the “back-to-back” translation rule. This ensured the preservation of the content and meaning of the data. Next, the transcripts were methodically coded by the first author, after which, the codes were organized under deducted themes. The consolidated criteria for reporting qualitative research (COREQ) were followed for this report (37).

## Results

### Background characteristics

A total of 27 participants took part in the study. Table 1 shows the distribution of age, gender, professional role, work experience, job title and qualification of participants in the study. The age of participants ranged between 23 and 63 years, of whom half were between 25 and 34 years of age. Most participants were female (*n* = 25), which reflects the current predominance of female health professionals in Ghana. The professional experience of participants ranged from 2 to 35 years and most participants had worked for more than 8 years (*n* = 20).

**Table 1:** Background characteristics of participants.

**Characteristic**	**Frequency**
Age group (years)	
30–35 36–40 41–45 46–50 51 and above Total	6 4 8 3 6 27
Gender	
Male Female Total	2 25 27
Work experience (years)	
< 5 5–10 >10 Total	3 10 14 27
Professional category	
General nurse Community health nurse Public health nurse Social worker Nurse manager Total	8 11 5 2 1 27

**Figure 1:**
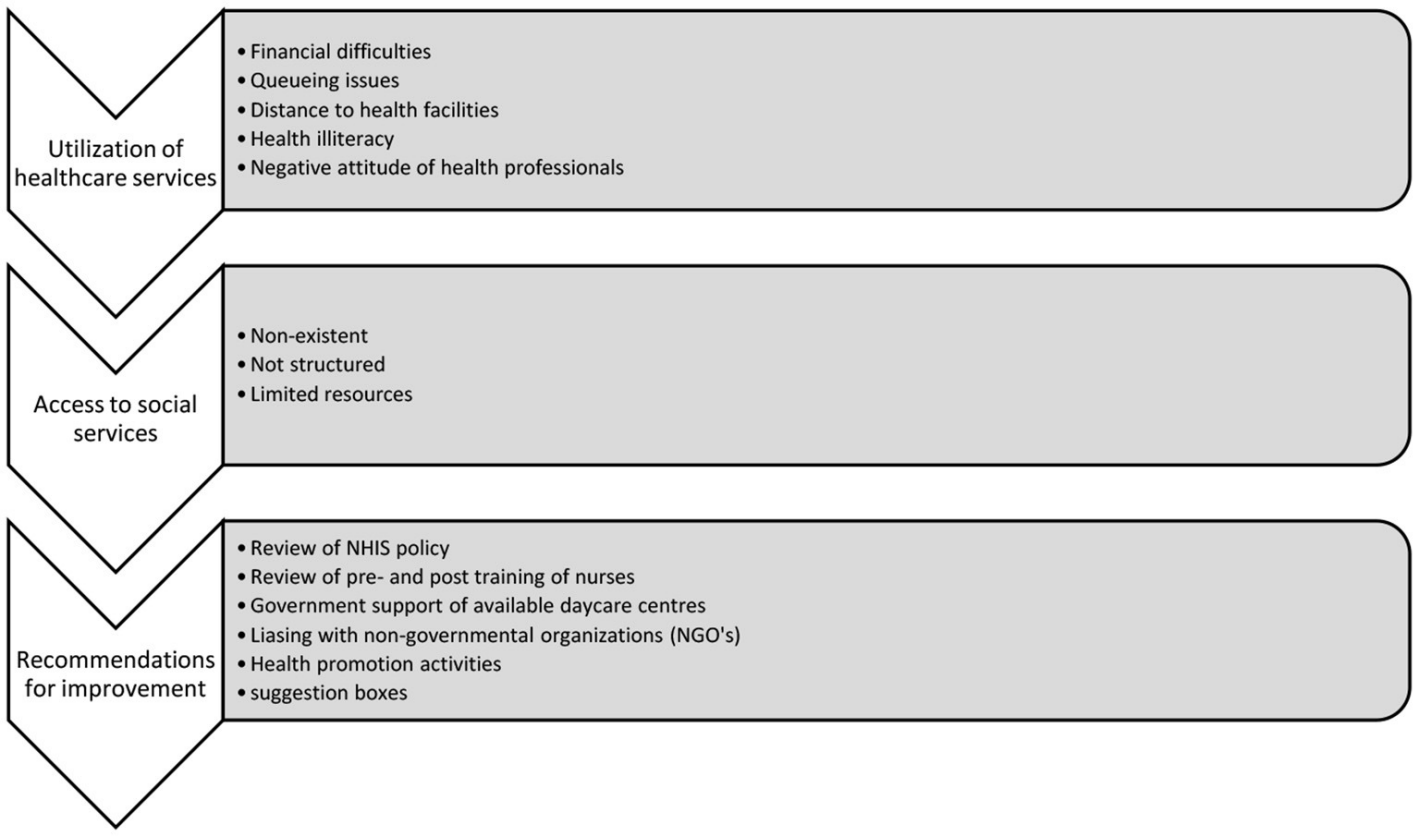
Chart showing themes and subthemes.

All interviews lasted between 50 min and 1 h. Data saturation was reached after one FGD with 10 participants and 17 individual interviews (as described in the Methods Section, the FGD appeared to not be the best method to get an in-depth understanding of the theme and therefore individual interviews were performed). In the analysis, three themes and fourteen subthemes were conceptualized. This process is detailed in Figure 1 below.

### Utilization of healthcare services

Participants discussed their perspectives on the reasons for the under-utilization of healthcare services among older adults living in slums. From these discussions, five themes emerged. The theme most often mentioned (by all 27 participants) was “financial difficulties”. This was followed by “queues” (*n* = 25), “health illiteracy” (*n* = 24), and “unwelcoming health professionals” (*n* = 20, mostly nurses). Lastly, “proximity of health facilities” to the slums was mentioned by some participants as a factor influencing the utilization of healthcare services. Below, the themes are described in more detail, including supporting quotes.

#### Financial difficulties

Health professionals mentioned that most slum-dwelling older adults face financial challenges and are not able to access the health facility for this reason. They explained that although some older adults have registered with the National Health Insurance Scheme (NHIS), most do not have an active card or are not registered at all. Besides, even with an NHIS registration, additional payments must be made. The narratives of 2 participants exemplify the theme “financial difficulties”.

“*… They pay for some of the services they receive at the hospital even with the introduction of the NHIS Card and this should not be. They are suffering especially financially…”* (PHN2).

“*Most of them do not have health insurance. … so they find it difficult going to the hospital because they have to make payment for everything”* (CHN11).

#### Queues

Participants mentioned long waiting times at the health facility as an important reason to not access healthcare services. Professionals argued that the processes patients go through before seeing the doctor can be stressful for older adults. Because there are no special units for older adults, they are seen by the same doctors attending to the younger adults. Participants who work in the hospital said they sometimes select the patients and create a different queue for older adults although not every health professional does this.

“*…when they come to the facility, they have to go to records first and search for their folders (*Patients records*) and it takes a long time before they come to have a physical assessment done and then be seen by a doctor… the process is tedious for them…”* (RGN 4).

“*Although there are no special units for old people, we sometimes separate them from the young ones to minimize the waiting time…”* (RGN 1).

#### Health illiteracy

Participants discussed that not every old slum-dwelling adult was illiterate. However, health illiteracy remains a problem which influences the use of formal health services. Participants observed that most older adults in the slums attribute every ill-health to spiritual factors and will not usually use formal care services. They argued to see that some older adults receive medication from the hospital but do not adhere to the treatment regimen because of preferences for herbal preparation.

“*I cannot vividly say yes, and I cannot say no, … in the setting in which we live, superstition supersedes a lot of things… most of them will rather use herbal medicine…”* (CHN 9).

#### Unwelcoming health professionals

Health professionals mentioned their attitude toward older slum-dwellers. They stated that older adults from slums were not treated with a lot of respect. Nurses were the health professionals mostly accused. Participants attributed the unwelcoming nature of most nurses to work challenges as well as personal problems.

“*Well, I can say that during our training we did not receive much education on caring for older adults. So, the skills in attending to older adults may be missing… Also, especially at the OPD* (Outpatient Department)*, the patients can be many whilst staff on duty are few so they may be overwhelmed…”* (RGN 6).

“*Our attitude … it's zero I won't even say one because if an old lady comes to the hospital and you being the health worker, for example, cannot give the older person priority care, … some of our people (*nurses) *are very rude. We all have our frustrations, but you should not displace them on the poor old patient… So, for our attitude, it is nothing to write home about”* (RGN 2).

#### The proximity of health facilities

Health professionals reported that poor quality of roads and uneven walkways, as well as the distance from the slum to the health facility influence utilization of health facilities.

“*The poor road network and many untarred roads in the slums predispose the old people to falls so they would not be comfortable walking. Also, the place is far but there is no money for transportation. So, they stay at home and combine herbal mixtures till the complications begin to surface”* (CHN 9).

### Access to social services: Non-existent, unstructured, and limited resources

Under the main theme ‘access to social services', three subthemes emerged: social services being non-existent, social services being unstructured, and limited resources in available services to cater for all older adults in nearby slum communities.

Most health professionals (n=24) mentioned that social care services were non-existent in the slum communities nearby.

“*A recreational place for old people? I have not heard of any around here…in the slums, everyone is thinking about the children* (playground) *and the government has not set up anything for the old people”* (PHN 5).

A few health professionals (*n* = 3) described a social care service but also described that this service did not have a guiding structure (schedule).

“*There is a big tree in the community across the street. I know the old people go and sit there and have conversations. Sometimes we* (nurses) *gather them* (old people) *and give them a health talk. But they don't have any structured organization for their activities…”* (CHN 8).

Participants from the aged care centers stated that even though they provided some social care services such as local indoor games, health screening and health education to older adults from slum communities, resources were not available to make it freely and widely accessible.

“*In our place, we accept the old people within the vicinity three times a week. We give them health talks, engage them in local games like Ludu and Oware* (local games with dice and pebbles) *and sometimes we allow them to tell stories. We feed them breakfast and lunch provided by benevolent people… However, because of the limited resources, we cannot transport people to the center so only the few who are nearby will attend…it's sad”* (SW2).

### Recommendations for improvement

Under the main theme recommendations for improvement, six subthemes were identified: (1) review of NHIS policy, (2) review of pre-and post-training of nurses, (3) government support with available day-care centers, (4) liaising with non-governmental organizations (NGOs), (5) health promotion activities, and (6) placement of suggestion boxes around health facilities.

#### Review of NHIS policy

All participants suggested a review of the NHIS policy to make healthcare free for people 60 years and above. They also stated that including treatment for more disease conditions common among the aged population is essential.

“*The NHIS policy should be reviewed if we want to improve the health and overall QoL of our older people especially those in the slums. They don't have the money so at least if they are registered free with no renewal costs, it will be a big relief! … they should also review the health conditions covered by the NHIS to include diseases of old people …”* (CHN 9).

#### Review of pre-and post-training of nurses

All nurses in this study spoke about the current training of nurses and lamented that a lot of nurses are being trained, but the quality of professionalism among nurses is questionable because of their approach to health care. They added that training should result in intrinsic motivation and a professional attitude to care for older adults.

“*The best way to curb and improve on this* [unwelcoming/unfriendly professionals] *is to encourage students in our various nursing training institutions to have the desire to care for people and also make gerontology an interesting course for nursing students to appreciate…”* (NM1).

“*Training of students on not stigmatizing old people will possibly improve their attitude when they start practicing. Having regular workshops, post-training, on avoiding discriminatory actions toward old people in the slums may perhaps help”* (RGN 3).

#### Government supporting available daycare centers

Participants who were from the aged care centers advocated for governmental support so that services can spread to a wider catchment area.

“*…Yes, we are trying our best with the little resources…if the government can factor us in their budget planning and help us financially, there will also be enough to care for older adults in the slums. A bus to transport people will be appreciated also…”* (SW 1).

#### Liaising with non-governmental organizations (NGOs)

Health professionals also suggested that NGOs and private individuals should collaborate and set up centers in communities to provide social services.

“*…in the demarcated constituencies in every region, at least there should be one center where social services can be provided for old people. Just like playgrounds have been set up for the children, NGOs can also do something for the old people in the slum communities….”* (PHN 3).

#### Health promotion activities

All participants expressed that health promotion activities and free health screening to prevent and detect any health issues should be held frequently in the slums for older adults and their informal caregivers. They hoped that doing this will improve the health literacy of the slum-dwelling older adult while bringing health services to the doorsteps as the hospitals are far and the roads are not the best for the older adults. The narrative below by RGN 6 summarizes the perspective of all participants.

“*…I will say that if we carry out health education and health promotion activities more often in the slums, we can improve the health literacy of the older adults…and also get closer to them…”* (RGN 6).

#### Suggestion boxes

Some health professionals working in the 2 hospitals suggested placing suggestion boxes in the compound of health facilities which should be regularly checked for patient complaints, especially regarding staff attitude.

“*Suggestion boxes at vantage places will also be very helpful. Now it is compulsory to wear your name tag on duty so patients can easily identify us. Now patients can name health professionals who are rude and unprofessional … so that authorities can take appropriate action…”* (RGN 4).

## Discussion

Previous studies have indicated general barriers to health care services however this study specifically focused on barriers for older slum-dwellers. This study explored the views of health professionals on the access to and use of health and social care services among older slum-dwelling adults and their recommendations for improvement. The participants in the study stated that slum-dwelling older adults' access to, and use of health and social care services was influenced by financial barriers, queues to access care services, the attitude of health professionals, long distance to health facilities, health illiteracy, and unavailability of formal social care services. All healthcare professionals interviewed commented that healthcare and social services for older adults need improvement. The general recommendations for improving health and social care services for older adults in slums that emerged from this study were: (1) review of NHIS policy to include free healthcare for adults aged 60 years and above, (2) review of pre-and post-training of nurses on geriatrics, (3) government support for day care centers for older adults, (4) liaising with non-governmental organizations (NGO's) to provide formal social care services and free registration of the health insurance, (5) health promotion activities by health professionals in slum communities, (6) provision of suggestion boxes around health facilities for compliments and complains.

The barriers to health care (financial challenges, health illiteracy, long distances to services, long waiting times in healthcare facilities and poor attitude of health professionals) described by participants in this study are not new, as they were also found in earlier studies performed in Ghana mentioned by Braimah and Rosenberg (38) and Wuaku et al. (39), in India by Chauhan and Saxena (40) and in Thailand by Jirathananuwat (41).

Health professionals in this study stated that older adults in Ghanaian slums were aware they needed to pay for their health insurance cards as well as yearly renewals, but that they were financially unable to do so. This finding agrees with a study by Amiresmaili et al. (21) in Iran (which is comparable to Ghana as it is an emerging lower-middle-income country), where lack of financial support was a major barrier because of the low economic status of the slum-dwelling older adult in Ghana. As postulated by Amiresmaili et al. (21) reducing financial and non-financial barriers to accessing healthcare is likely to encourage the utilization of healthcare services. Recommendations of health professionals in this study regarding a review of the current NHIS policy (to ensure free healthcare for older adults 60 years and above instead of the current age range of 70years and over) resonate with previous studies in Africa which suggested that better insurance coverage improved access and use of healthcare services (18, 38, 39, 42).

From the focus group discussion, it emerged that the poor attitude of health professionals is also a barrier to the utilization of healthcare services by older adults in slums. Wang et al. (43) in their study of attitudes of Community health professionals also reported negative attitudes toward dementia patients which affected healthcare use in Changsha, China. This confirms a scoping study on low- and middle-income countries by Sarikhani et al. (44) which suggested that attitudinal barriers affect the utilization of healthcare services. Health professionals in this study recommended a review of the pre-and post-training of nurses to improve their attitude toward older adults in slums and make them more welcoming. This agrees with the recommendation by Wang et al. (43) that providing opportunities for education and training will be beneficial in improving the attitude of community health professionals and with more recent studies by Amsalu et al. (45) in Ethiopia, Tsiga-Ahmed et al. (46) in Nigeria and Tavares et al. (47) in Portugal.

Queueing at health facilities by older slum-dwellers was cited as a barrier to healthcare service access and use. This confirms previous studies by Oche et al. (48), Chauhan and Saxena (40), and Naz et al. (49) from Nigeria, India, and Pakistan, respectively. From these studies, older adults resorted to self-medication from pharmacies as this was an easier option compared to visiting the hospital. In more developed countries options for telehealth are being explored (50) but considering the literacy and economic level of older adults in Ghanaian slums, this will be a difficult initiative (but not impossible) to implement in this setting.

Lastly, the literature suggests that social care provided by family members is commonly seen in most African countries (51–53), moreover, from this study, formal social care services appeared to be next to non-existent (34, 54–56).

### Strengths and limitations

A strength is that this study is the first of its kind which has explored the views of health professionals on improving health and social care services for slum-dwelling older adults in Ghana. The findings of this study can be used to guide the design and formulation of policies that aim at removing barriers to formal health and social care use among older adults in Ghanaian slums. A possible limitation of this study is the absence of physicians/medical doctors and other health professionals from the participants. These professionals were approached but they declined, stating they were unavailable or uninterested. Therefore, it is recommended for future studies to think of incentives that would make medical doctors and other health professionals interested to participate in such studies. Additionally, having a small number of participants from social services is a limitation, which makes external generalizability limited. Future studies should include more social workers as participants to provide more suggestions for better interventions to meet the social care needs of older adults in slums.

### Implications for practice

This study stresses the need for health professionals in Ghana to change their attitude toward slum-dwelling older adults, which might be achieved through regular post-training workshops at the health facilities. Additionally, as health illiteracy is a contributing factor to low accessibility and use of healthcare services, this study encourages community and public health nurses to conduct activities aimed at improving health literacy among older adults in slums. A method could be health education at durbars in the communities or home visits to the older adults to interact with them. Lastly, health professionals in this study, are advocating for the government to support available day-care centers in Ghana to provide resources for the provision of social care services in these centers.

### Implications for further research

Based on the results of this study, future research should focus on what social care services could improve the quality of life for slum-dwelling older adults. It is recommended to involve more social workers in these studies. Additionally, we recommend interventional studies to see if some of the practical strategies suggested in this study, can indeed remove some of the barriers intended to address. An example could be the post-training of nurses to address attitudinal barriers or health screening and health education to improve health literacy among older adults in slums. Lastly, similar studies can be replicated in other big regions of the country to ascertain any differences or similarities in the findings.

## Conclusion

This study found barriers to health and social care access and use among slum-dwelling older adults, according to the perspective of health professionals. Addressing these barriers is essential to diminish the negative influence on their utilization of formal health and social care services and health inequity. Furthermore, the health professionals participating in this study suggested practical strategies to overcome these barriers, such as providing free healthcare for adults 60 years and over as well as ensuring a positive attitude of health professionals. It is hoped that these recommendations if adhered to, will improve the health and social care which is given to older adults.

Additionally, slum reforms and policymakers can implement these recommendations to help meet the health and social needs of older adults in slums. To be able to reach the goal of the Universal Health Coverage policy (12, 57) of “ensuring that all people have access to needed, quality health services without suffering financial hardship”, Ghana needs to aim at improved healthcare for its population.

## Data availability statement

The original contributions presented in the study are included in the article/supplementary material, further inquiries can be directed to the corresponding author/s.

## Ethics statement

The studies involving human participants were reviewed and approved by Institutional Review Board 37 Military hospital. The patients/participants provided their written informed consent to participate in this study.

## Author contributions

PA, IE, CL, AA, and JS conceptualized the study. PA conducted the interviews. PA and IE analyzed the data. All authors reviewed the sections Introduction and Discussion and read through the final manuscript before submission. All authors contributed to the article and approved the submitted version.

## Conflict of interest

The authors declare that the research was conducted in the absence of any commercial or financial relationships that could be construed as a potential conflict of interest.

## Publisher's note

All claims expressed in this article are solely those of the authors and do not necessarily represent those of their affiliated organizations, or those of the publisher, the editors and the reviewers. Any product that may be evaluated in this article, or claim that may be made by its manufacturer, is not guaranteed or endorsed by the publisher.

## Abbreviations

FGD: Focus Group Discussion
